# Rectal Stricture Caused by a Rare Plasmacytoid Urothelial Carcinoma

**DOI:** 10.1155/2024/4823396

**Published:** 2024-05-24

**Authors:** Ahmad Alnasarat, Rasheed Musa, Asaiel Makahleh, Mohammad N. Kloub

**Affiliations:** ^1^Michigan State University, Lansing, MI, USA; ^2^Department of Internal Medicine, Division of Gastroenterology, Hepatology and Nutrition, East Tennessee State University, Johnson, TN, USA; ^3^Department of Internal Medicine, East Tennessee State University, Johnson, TN, USA; ^4^Department of Internal Medicine, Saint Micheal's Medical Center, Newark, NJ, USA

## Abstract

Malignant rectal strictures are uncommon, but they may pose a diagnostic challenge in clinical practice. We report the case of an 85-year-old male with an initially puzzling presentation of abdominal distention and discomfort. The patient was ultimately diagnosed with a rectal stricture caused by a plasmacytoid variant of urothelial cell carcinoma originating from the bladder. This case emphasizes the necessity of considering unique etiologies when evaluating rectal strictures and the aggressive character of this type of urothelial carcinoma.

## 1. Introduction

Bladder cancer is the 4^th^ most common cancer in men and 8^th^ most common in women, primarily originating from urothelial cells [1]. Invasion of the rectum is uncommon, possibly due to the belief that metastatic spread would not penetrate the muscular layers of the gastrointestinal tract, thereby preserving the rectal mucosa's integrity [2]. The plasmacytoid variant of urothelial cell carcinoma is a particularly rare and aggressive subtype of bladder cancer that can extend beyond the bladder, leading to unexpected presentations such as rectal strictures [3]. We report on the case of a rectal stricture caused by a plasmacytoid variant of urothelial cell carcinoma after presenting with a nonspecific picture. This case highlights the importance of considering uncommon etiologies in evaluating rectal strictures.

## 2. Case Report

An 85-year-old male with no significant medical history presented with complaints of epigastric discomfort and abdominal distention that had developed only three days prior to presentation. He reported an unintentional weight loss of approximately 25 pounds over the preceding three months. The patient denied smoking, alcohol consumption, peptic ulcer disease, nonsteroidal anti-inflammatory drug (NSAID) use, or family history of gastrointestinal malignancy or genetical diseases. He had never undergone previous esophagogastroduodenoscopy (EGD) or colonoscopy. The patient had normal bowel movements four days before presentation and was passing flatus.

Physical examination revealed abdominal distension, mild epigastric tenderness, and a tympanic note on percussion, with no evidence of an acute abdomen. Bowel sounds were sluggish, and the patient declined a digital rectal examination.

EKG showed normal sinus rhythm with a pulse of 88. Initial workup results showed white blood count 6.3 K/cumm, Hb 12.8 g/dL, platelets counts of 305 K/cumm, Na^+^ 137 mMol/L, K^+^ 3.9 mMol/L, BUN 21 mg/dL, Cr 0.86 mg/dL, total bilirubin 0.51 mg/dL, AST 23 Units/L, ALT 13 Units/L, ALP 89, total protein 7.2, albumin 3.8 gm/dL, lipase 3 Units/L, and troponin 3 ng/L. CT abdomen showed thickening of the distal esophagus, marked gastric dilation with gas and fluid contents, and a large stool burden, suggesting gastric outlet obstruction.

A nasogastric tube was placed for decompression, and the patient received lactulose and polyethylene glycol-based lavage solution (Golytely) to relieve constipation, resulting in partial symptom relief. EGD was performed the following day and was unremarkable. The patient tolerated diet advancement but experienced recurrent abdominal distension and discomfort two days later. Repeat CT of the abdomen and pelvis demonstrated small and large bowel distension and nonspecific.

**Figure 1:**
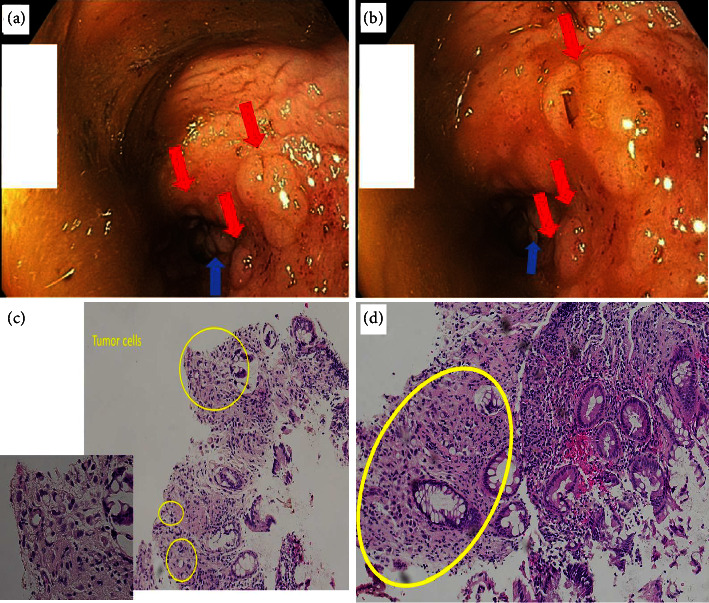
(a, b) Endoscopic view of the rectum showing multiple submucosal nodules along with rectal stricture. Red arrow points to submucosal nodules. Blue arrow points to the stricture area. (c) Tumor cells and unremarkable colonic glands. (d) Rectal biopsy showing unremarkable surface mucosa with focal poorly differentiated infiltrating carcinoma in the lamina propria with a signet ring component.

Circumferential rectal wall thickening. Flexible sigmoidoscopy revealed a 5 cm long rectal stricture surrounded by multiple submucosal nodules with no identifiable mucosal masses (Figure 1). Biopsies were obtained, and pathology results indicated a plasmacytoid variant of urothelial cell carcinoma Figure 1.

**Figure 2:**
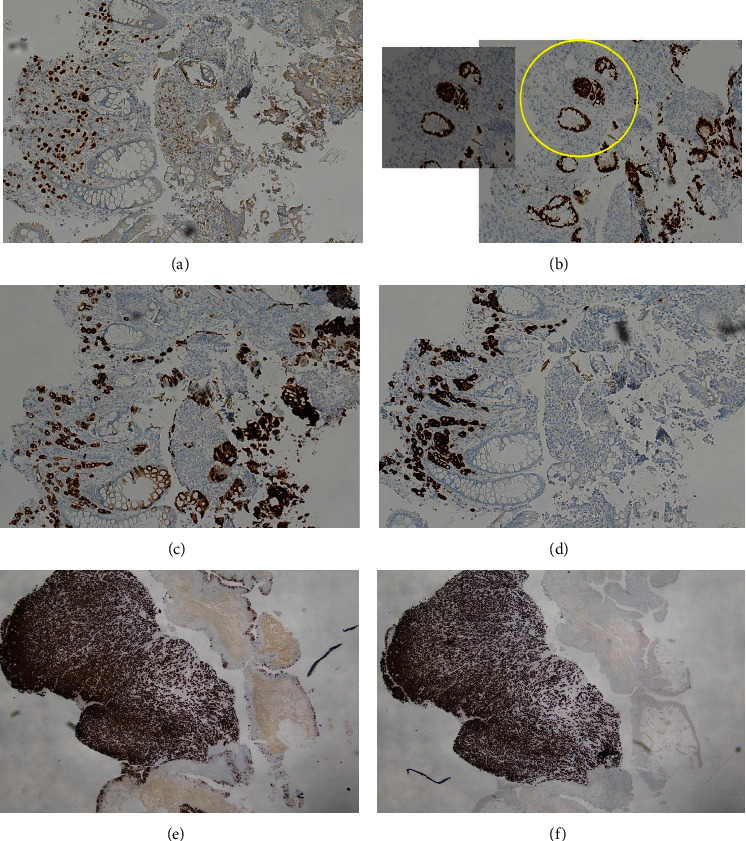
(a) GATA positive. Consistent with rectal involvement by urothelial carcinoma (b) CDX2 negative in tumor with normal staining of colonic glands (c) CK20 positive (d) CK7 positive. (e, f) CK7 and CK20 positive. Typically coexpressed in urothelial carcinoma.

These were confirmed by the immunohistochemistry profile given in Figure 2.

**Figure 3:**
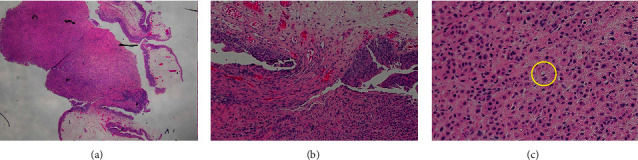
(a) Bladder biopsy, urothelial carcinoma, plasmacytoid/signet ring/diffuse variant. The tumor involves lamina propria with polypoid cystitis in the overlying urothelium. No surface urothelial dysplasia. (b) Transition of polypoid cystitis (upper) to tumor (lower). (c) The tumor consists of a discohesive population of single cells with eccentric nuclei, eosinophilic cytoplasm, and occasional signet ring forms (H&E stain 200x) (circle).

Cystoscopy revealed nonspecific bladder wall thickening, and biopsies confirmed the same pathology as that was found in the rectum Figure 3. The patient's clinical condition deteriorated rapidly leading to referral to palliative/hospice care, and then the patient passed away a few days later.

## 3. Discussion

The plasmacytoid variant of urothelial cell carcinoma is a rare and aggressive subtype of bladder cancer characterized by plasmacytoid or signet-ring cell morphology [4]. This variant is associated with a higher risk of metastasis and an overall poor prognosis [4]. It can extend beyond the bladder, involving adjacent organs, such as the rectum, leading to unexpected clinical presentations like rectal strictures [4] or distant metastatic like stomach [5]. The exact process by which urological malignancies cause rectal stricture is unknown. However, numerous studies propose different possibilities on the pathways by which invasive bladder cancer may spread to the rectum. These explanations include deposition as a result of past iatrogenic exposure, such as surgically introducing cancer cells. Lack of previous pelvic surgery makes it unlikely in our case. Direct invasion in which the tumor enters the bladder wall via Denonvilliers' fascia and advances to the rectum, or metastasis from the bladder's lateral pedicles to the posterior rectal wall, eventually leading to wall infiltration [6], these could apply to our patient.

The poor outcomes and delayed presentation could be attributed to the absence of urological symptoms and lack of identifiable bladder mass [7, 8]. Similar to our case, cystoscopy typically reveals thickening of the bladder wall without a defined mass [7, 8], which adds to the challenge of diagnosing such a disease. Malignant rectal strictures often present with symptoms such as abdominal pain, constipation, or obstructive symptoms[6]. Diagnosis typically involves endoscopic evaluation with biopsy to determine the underlying malignancy [6]. Management depends on the extent of the disease, the patient's overall health, and the aggressiveness of the malignancy [9].

Although there is no well-defined management of plasmacytoid urothelial carcinoma, data suggest radical cystectomy and adjuvant chemotherapy due to advanced disease in most cases [7]. Since our patient's diagnosis was delayed, preventing him from receiving curative treatment before his disease became metastatic and palliative/hospice care was considered, in light of the limited availability of curative treatment options and the poor prognosis [9]. Further studies are required in the future to define appropriate treatment strategies for this condition.

In our case, we concluded that a secondary tumor in the rectum might be the only sign of concealed bladder cancer, especially in the absence of urological symptoms. This requires a high index of suspicions as the symptoms exhibited were nonspecific and could resemble those of other gastrointestinal conditions, such as gastric outlet obstruction, dyspepsia, Ogilvie syndrome, stool impaction, or intestinal obstruction. Laboratory assessment ruled out any possible causes that might contribute to such a clinical picture like hypokalemia or thyroid diseases. CT imaging, EGD, and colonoscopy were conducted to investigate the etiology. However, despite these diagnostic efforts, the underlying cause remained unknown until pathology report results were received.

## 4. Conclusion

Although malignant rectal strictures are rare, it can result from various underlying etiologies, including urothelial cell carcinoma of the bladder, especially its plasmacytoid variant. This variant is rare and aggressive and requires a high index of suspicion to ensure a proper diagnosis, as a timely diagnosis can significantly impact patient management and prognosis. Recognition of this aggressive subtype of bladder cancer and its potential to involve adjacent organs, such as the rectum, is crucial for appropriate evaluation and treatment.

## Data Availability

The data used to support this study are included in the article. Further inquiries can be directed to the corresponding authors.
